# A Systematic Review and Meta‐Analysis of the Association Between Obesity and Albuminuria in Asian People

**DOI:** 10.1155/ijhy/5660680

**Published:** 2026-09-02

**Authors:** Maria Riastuti Iryaningrum, Louis Fabio Jonathan Jusni, Rivaldi Ruby, Ria Bandiara, Yunia Sribudiani, Nanny Natalia Mulyani Soetedjo, Rudi Supriyadi

**Affiliations:** ^1^ Doctoral Study Program in Medical Science, Faculty of Medicine, Padjadjaran University, Bandung, Indonesia, unpad.ac.id; ^2^ Department of Internal Medicine, School of Medicine and Health Sciences, Atma Jaya Catholic University of Indonesia, Jakarta, Indonesia, atmajaya.ac.id; ^3^ School of Medicine and Health Sciences, Atma Jaya Catholic University of Indonesia, Jakarta, Indonesia, atmajaya.ac.id; ^4^ Department of Internal Medicine, Faculty of Medicine, Hasan Sadikin General Hospital, Padjadjaran University, Bandung, Indonesia, unpad.ac.id; ^5^ Department Biomedical Sciences, Division of Biochemistry and Molecular Biology, Faculty of Medicine, Hasan Sadikin General Hospital, Padjadjaran University, Bandung, Indonesia, unpad.ac.id; ^6^ Department of Internal Medicine, Division of Endocrine and Metabolism, Faculty of Medicine, Hasan Sadikin General Hospital, Padjadjaran University, Bandung, Indonesia, unpad.ac.id; ^7^ Department of Internal Medicine, Division of Nephrology and Hypertension, Faculty of Medicine, Hasan Sadikin General Hospital, Padjadjaran University, Bandung, Indonesia, unpad.ac.id

**Keywords:** albumin–creatinine ratio, albuminuria, meta-analysis, obesity, sian

## Abstract

Obesity‐related renal dysfunction is largely attributed to hemodynamic alterations and insulin resistance. Among individuals with overweight or obesity, albuminuria has been shown to be independently and inversely associated with circulating adiponectin levels in the absence of diabetes. Accordingly, this study aimed to evaluate and quantify the independent association between obesity and the risk of kidney disease, as assessed by the presence of albuminuria in the Asian Population. This systematic review was conducted in accordance with the Preferred Reporting Items for Systematic Reviews and Meta‐Analyses (PRISMA) guidelines. A comprehensive and systematic search strategy was applied across multiple electronic databases, including PubMed, ProQuest, Taylor & Francis, and Wiley, to identify relevant studies meeting the eligibility criteria. Data synthesis and statistical analyses were carried out using Review Manager (RevMan) software Version 5.4, and the methodological rigor of the included studies was independently assessed using the Newcastle–Ottawa Scale (NOS). The pooled analysis demonstrated that obesity was significantly associated with albuminuria in the general population as well as in sex‐specific subgroups. Specifically, the odds of albuminuria were higher among individuals with obesity in the overall population (OR = 1.76; 95% CI: 1.09–2.83; *p* = 0.02, *I* ^2^ = 78%), in men (OR = 1.34; 95% CI: 1.05–1.71; *p* = 0.02, *I* ^2^ = 63%), and in women (OR = 1.16; 95% CI: 1.05–1.28; *p* = 0.004, *I* ^2^ = 19%). Obesity was associated with albuminuria in Asian Populations.

## 1. Introduction

Obesity is widely recognized as a chronic metabolic disorder characterized by excessive fat accumulation as its core pathophysiological feature [1]. Its development is driven by complex biological mechanisms interacting with an increasingly obesogenic environment, contributing to the rising global prevalence and incidence [2]. Current projections estimate that obesity rates will increase by 50% between 2025 and 2030, affecting nearly 3 billion adults worldwide. Asia, in particular, accounts for more than one‐fifth of the global obesity burden. The growing prevalence of obesity is accompanied by a surge in related health complications. In 2021, noncommunicable diseases (NCDs) were responsible for approximately 10.7 million premature deaths globally, with 15% linked to elevated body mass index (BMI) [3]. Obesity‐associated mortality is closely related to conditions such as type 2 diabetes mellitus (T2DM), hypertension, nonalcoholic fatty liver disease, cardiovascular disorders including myocardial infarction, heart failure, atrial fibrillation, stroke, obstructive sleep apnea, and renal impairment [4].

Renal complications in obesity are largely attributed to hemodynamic alterations and insulin resistance [5]. Hypertension and T2D, as major consequences of obesity, further contribute to the decline in kidney function [6]. More recently, obesity itself has been identified as an independent risk factor for chronic kidney disease (CKD), largely mediated by adipotoxic mechanisms [7]. Chang et al. reported that increased BMI, waist circumference (WC), and waist‐to‐height ratio (WHR) are independent predictors of reduced glomerular filtration rate (GFR) and higher mortality risk, both in individuals with existing kidney disease and in those without prior renal pathology [8]. Among overweight and obese individuals without T2DM, albuminuria has been shown to have an inverse relationship with circulating adiponectin levels [9]. Lower adiponectin concentrations are linked to disrupted fatty acid metabolism and the progression of insulin resistance, chronic inflammation, and oxidative stress [5]. Experimental studies in animal models further demonstrate that adiponectin deficiency can lead to podocyte damage, interstitial fibrosis, and increased urinary albumin excretion [10].

Several studies have highlighted the association between obesity and kidney dysfunction, particularly albuminuria. Evidence from Chinese and Korean populations indicates that individuals with obesity have approximately twice the risk of developing albuminuria compared with nonobese individuals [11, 12]. Despite this evidence, there remains a lack of clear guidelines or standardized quantitative measures defining obesity‐related kidney disease. Therefore, this systematic review and meta‐analysis aims to evaluate and quantify the independent association between obesity and kidney disease, as assessed by the presence of albuminuria.

## 2. Method

This systematic review was planned and carried out in line with the 2020 PRISMA guidelines [13]. The study protocol was prospectively registered in the PROSPERO database under the registration number CRD420261283414.

### 2.1. Eligibility Criteria

**Types of studies:** This review included both published and unpublished observational research exploring the association between obesity and albuminuria in adults. To maintain methodological rigor and relevance, several study designs were excluded, including randomized controlled trials, systematic and narrative reviews, case reports, case series, conference abstracts, book chapters, editorials, and commentaries, as well as animal studies. Articles not written in English or without accessible full texts were also excluded. These criteria ensured that only relevant, high‐quality, and accessible evidence was analyzed, as summarized in Table 1.

**TABLE 1:** PICOTS‐SD criteria.

Patients	Asian adult patients aged > 18 years
Intervention or exposure	Adults were diagnosed with obesity based on BMI, WC, WHR, and imaging
Comparator	Adults without general obesity, visceral obesity, or central obesity
Outcomes	Albuminuria (uACR > 30 mg/g)
Time	No publication time restriction
Setting	Patients visiting medical facilities
Study design	Observational studies (cohort, case–Control, and cross‐sectional)

**Participants:** Adults aged 18 years and older were included in the study. Obesity was classified into peripheral, central, and visceral obesity. Peripheral obesity was assessed using BMI, whereas central obesity was evaluated using WC and WHR. Visceral obesity was determined by measuring the visceral adipose tissue (VAT) cross‐sectional area using a 16‐detector‐row computed tomography (CT) scanner. All diagnostic criteria for obesity were summarized in Table 2. Participants were excluded if they had confirmed CKD, diagnosed based on clinical evaluation, imaging findings, laboratory tests, and/or histopathological examination, with a disease duration of more than 3 months. Individuals with other kidney diseases associated with increased urinary protein excretion, including primary or secondary nephropathies, were also excluded. Additional exclusion criteria included the daily use of angiotensin‐converting enzyme (ACE) inhibitors or angiotensin II receptor blockers (ARBs), incomplete or missing essential study data, hematuria detected by dipstick urinalysis, active infection, or a diagnosis of cancer.

**TABLE 2:** Characteristics of included studies.

No	Author, publication year, country	Study design	Population						Obesity parameter	Adjusted confounder	Inclusion criteria	Outcome of interest		
			Total		Gender		Age (mean ± SD)/(median (SD))					Odds ratio		
			Obesity	Control	Obesity	Control	Obesity	Control				Total	Men	Women
1	Shaw, et al. 2007, South Asian	Cross‐sectional	205		M: 90 F: 115		37.3 ± 9.4		Central obesity (WHR≥ 0.90 in men and ≥ 0.85 in women)	WHR, sex, age, smoking, BMI, blood pressure, CRP, and glucose	A total of 205 adult participants of South Asian origin were enrolled in this study. All included subjects were normoglycemic.	4.1	—	—
2	Lin, et al. 2012, Taiwan	Cross‐sectional	787	1563	M: 384 F: 403	M: 758 F: 805	59.2 ± 11.73	55.6 ± 11.	Central obesity (WC ≥ 90 cm for men and ≥ 80 cm for women)	Age, gender, BMI, systolic BP, diastolic BP, hsCRP, fasting glucose, eGFR, smoking, alcohol drinking, betel nut chewing, and physical activity.	This study included a total of 2350 participants, all of whom were residents of Taichung City, Taiwan, aged 40 years or older as of October 2004.	1.73	1.39	2.35
3	Kim, et al. 2014, Korea	Cross‐sectional	384	770	M: 261 F: 123	M: 523 F: 247	M: 56.4 ± 8.8 F: 54.9 ± 9.1	M: 54.9 ± 9.1 F: 54.6 ± 7.5	Visceral Obesity (VAT)	Age, sex, diabetes, hypertension, triglycerides, history of chronic kidney disease, aspirin medication, hormone replacement therapy, and smoking	A total of 1154 individuals who underwent routine comprehensive health examinations at the Healthcare System Gangnam Center of Seoul National University Hospital between September 2006 and December 2010 were included in the study. The participants ranged in age from 30 to 82 years.	1.96	2.74	1.70
4	Qin, et al. 2021	Cross‐sectional	South: 1873 North: 3143	South: 19,340 North: 16,729	South: M: 7046 F: 14.167		South: 58.26 ± 9.81		Peripheral Obesity and Central Obesity (WHR ≥ A00.90 in men and ≥ 0.85 in women, BMI≥ 28.0 kg/m^2^)	Gender, age, smoking, drinking, HbA1c, DBP, TG, and AST.	A total of 41,085 individuals from eight regional centers participating in the REACTION study (Risk Evaluation of cAncers in Chinese diabeTic Individuals, a longitudinal study) provided written informed consent. The northern centers included Dalian, Lanzhou, and Zhengzhou, while the southern centers comprised Guangzhou, Guangxi, Luzhou, Shanghai, and Wuhan. The study population consisted of permanent residents aged 40 years and older, who were randomly selected from three to five urban communities representing a mid‐level socioeconomic background.	1.14	North: 1.21 South: 1.39	North: 1.11 South: 1.16
					North: M: 5651 F: 14,221		North: 58.02 ± 8.65							

5	Nam, et al. 2024, Korea	Cross‐sectional	4979		M: 2243 F: 2736		M: 43.1 ± 0.5 F: 45.8 ± 0.5		General Obesity (BMI≥ 25.0 kg/m^2^, WC ≥ 90 cm for men and ≥ 85 cm for women)	Age, alcohol consumption, smoking status, physical activity, diabetes, hypertension, hypercholesterolemia, hypertriglyceridemia, eGFR, 25(OH)D, waist circumference, body mass index.	This study included a total of 4979 participants aged 18 years and older. The sample was selected based on geographic region, sex, and age using data from the 2005 National Census Registry to ensure representation of the entire noninstitutionalized civilian population of Korea.	—	0.49	1.45

**Outcome of interest:** Urinary albumin levels were measured using an immunoturbidimetric assay, and urinary creatinine was assessed through the Jaffe kinetic method. Albuminuria was defined as a urinary albumin‐to‐creatinine ratio (uACR) greater than 30 mg/g, obtained from a first‐morning spot urine sample, as presented in Table 1.

### 2.2. Search Strategy

A comprehensive literature search was conducted across multiple electronic databases, including PubMed, Wiley Online Library, Taylor & Francis, and ProQuest, to identify studies relevant to the research objective. The search was independently performed by two reviewers using predefined, database‐specific search strategies, which are presented in Table 3. For the PubMed database, the search strategy incorporated both Medical Subject Headings (MeSH) and free‐text terms to maximize retrieval of relevant studies. All identified records were exported to Mendeley reference management software, where duplicate citations were removed before the screening process. Titles and abstracts were independently screened by the two reviewers to assess eligibility. Any disagreements were resolved through discussion and consensus, with consultation from a third reviewer when necessary. Studies that met the initial screening criteria subsequently underwent full‐text assessment according to the predefined inclusion and exclusion criteria.

**TABLE 3:** Main keywords for the search strategy.

Databases	Keyword	Total studies
PubMed	(“Obesity”[Mesh] OR obesity[tiab] OR obese[tiab] OR overweight[tiab] OR “Body Mass Index” [Mesh] OR BMI[tiab] OR “Waist Circumference”[Mesh] OR “waist circumference”[tiab] OR “waist‐to‐hip ratio”[tiab] OR WHR[tiab] OR “visceral obesity”[tiab] OR “abdominal obesity”[tiab] OR “central obesity”[tiab]) AND (“Albuminuria”[Mesh] OR albuminuria[tiab] OR microalbuminuria[tiab] OR macroalbuminuria[tiab] OR “Urinary Albumin Excretion”[tiab] OR “urinary albumin”[tiab] OR “albumin creatinine ratio”[tiab] OR “albumin‐to‐creatinine ratio”[tiab] OR ACR[tiab] OR UACR[tiab]) AND (“Adult”[Mesh] OR adult∗[tiab]) AND (“Asia”[Mesh] OR Asia∗[tiab] OR Asian∗[tiab] OR China[tiab] OR Chinese[tiab] OR Korea[tiab] OR Korean[tiab] OR Taiwan[tiab] OR Taiwanese[tiab] OR Japan[tiab] OR Japanese[tiab] OR India[tiab] OR Indian[tiab] OR Indonesia[tiab] OR Indonesian[tiab] OR Malaysia[tiab] OR Malaysian[tiab] OR Singapore[tiab] OR Singaporean[tiab] OR Thailand[tiab] OR Thai[tiab] OR Vietnam[tiab] OR Vietnamese[tiab] OR Philippines[tiab] OR Filipino[tiab]) NOT (animals[Mesh] NOT humans[Mesh])	711
Taylor & Francis	(All: obesity) AND (All: albuminuria) AND (All: asian) AND (All: overweight) AND (All: body mass index)	302
Wiley	“obesity” anywhere and “albuminuria” anywhere and “overweight” anywhere and “body mass index” anywhere and “asian” anywhere and “microalbuminuria” anywhere	607
ProQuest	obesity AND overweight AND (body mass index) AND albuminuria AND microalbuminuria AND asian AND (albumin creatinine ratio)	249

### 2.3. Data Extraction

All included studies were reviewed in detail, and relevant information was systematically gathered, including the first author’s name, study design, sample size, participant demographics (age and sex), eligibility criteria, description of the intervention procedures, and the primary outcomes reported. Data extraction was conducted collaboratively by all members of the research team.

### 2.4. Quality Assessment

Prior to inclusion in the meta‐analysis, each study will undergo a quality appraisal using the Newcastle–Ottawa Scale adapted for cross‐sectional studies (NOS‐CS). This tool assigns a score from 0 to 10 across three domains: participant selection (up to 5 points), group comparability (up to 2 points), and exposure assessment (up to 3 points). Studies scoring 4 or below will be classified as low quality, those with scores of 5–6 as moderate quality, and those with scores of 7 or higher as high quality [14]. Articles deemed to be of low quality will not be included in the analysis. Two reviewers will independently conduct the NOS‐CS evaluation, and any differences in scoring will be resolved through discussion with a senior researcher.

### 2.5. Synthesis of Results and Statistical Analysis

The relationship between obesity and albuminuria was quantified using odds ratios (ORs) with corresponding 95% confidence intervals (CIs). Because the included studies differed in outcome definitions and measurement approaches, a random‐effects model was selected to accommodate interstudy variability and the possibility that effect sizes varied across settings. Combined estimates for comparisons were derived using the inverse‐variance technique.

Study heterogeneity was examined using the *I* ^2^ statistic. An *I* ^2^ value below 25% indicated low heterogeneity, 25%–50% represented moderate heterogeneity, and values exceeding 50% were considered high. When substantial heterogeneity was identified, sensitivity analyses were conducted to investigate potential sources. Statistical significance was determined using a two‐sided *p* value threshold of 0.05. For continuous outcomes, pooled effects were presented as weighted mean differences, with weights based on individual study sample sizes. All analyses were performed using Review Manager (RevMan) software, Version 5.4.

## 3. Result

**FIGURE 1:**
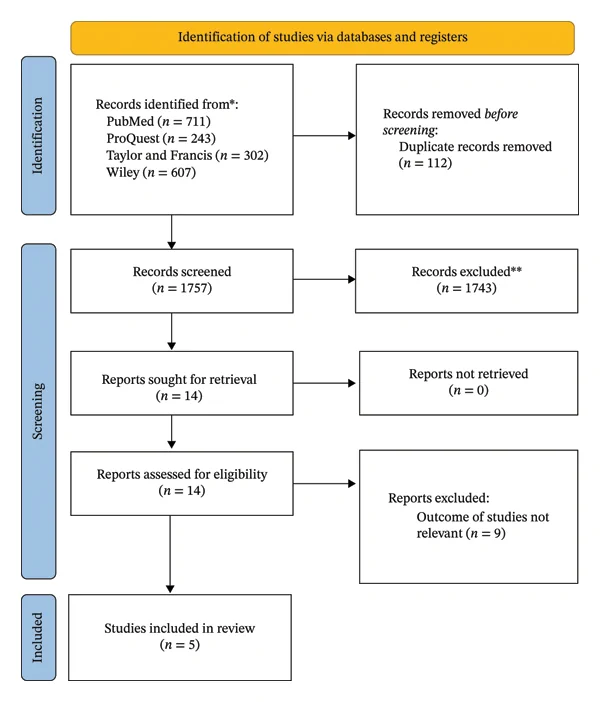
PRISMA 2020 flow diagram studies.

The study identification and selection process is illustrated in Figure 1. A comprehensive literature search across the selected databases identified 1757 potentially relevant records. After screening the titles and abstracts according to the predefined eligibility criteria, 14 articles were retrieved for full‐text review. Following detailed assessment, nine studies were excluded because their reported outcomes did not meet the predefined inclusion criteria. Ultimately, five studies fulfilled all eligibility criteria and were included in the systematic review and meta‐analysis.

### 3.1. Quality Assessment

The methodological quality of the included studies was assessed using the NOS‐CS, and the results are summarized in Table 4. Overall, the included studies were judged to have a low risk of bias. Most studies demonstrated good methodological quality; however, all studies except that of Lin et al. did not provide adequate justification for the sample size or report the response rate. In addition, the study by Shaw et al. did not use a representative sample of the target population. Based on the NOS‐CS assessment, the study by Shaw et al. received 7 stars, the study by Lin et al. received 10 stars, and the remaining studies each received 8 stars. The most common methodological limitations were the lack of sample size and response rate justification.

**TABLE 4:** Result of study quality assessment (NOS cross‐sectional).

Author, year	Selection				Comparability	Outcome		Score	Overall risk of bias
	Representativeness of the sample	Sample size	Nonrespondents	Ascertainment of exposure	Confounding controlled	Outcome assessment	Statistics	Total	AHRQ rating
Shaw, et al. 2007	0	0	0	^∗∗^	^∗∗^	^∗∗^	^∗^	7/10	Good (low)
Lin, et al. 2012	^∗^	^∗^	^∗^	^∗∗^	^∗∗^	^∗∗^	^∗^	10/10	Very good (low)
Kim, et al. 2014	^∗^	0	0	^∗∗^	^∗∗^	^∗∗^	^∗^	8/10	Very good (low)
Nam, et al. 2014	^∗^	0	0	^∗∗^	^∗∗^	^∗∗^	^∗^	8/10	Very good (low)
Qin, et al. 2021	^∗^	0	0	^∗∗^	^∗∗^	^∗∗^	^∗^	8/10	Very good (low)

*Note:* The asterisk (∗) indicates the scoring criteria for each domain and is used to facilitate the assessment of the Newcastle–Ottawa Scale (NOS).

### 3.2. Characteristics of Included Studies

A total of five cross‐sectional studies that met the established inclusion criteria were included into this systematic review, representing 49,773 participants overall. Essential study details, including sample size, age range, sex distribution, inclusion criteria, obesity parameters, potential covariates, study design, and measured outcomes, were carefully collected and presented in Table 2. The average age of participants across studies varied from 37.3 ± 9.4 to 59.2 ± 11.7 years, and females accounted for the majority of participants in these study populations [11, 12, 15–17].

### 3.3. Meta‐Analysis Results

**FIGURE 2:**
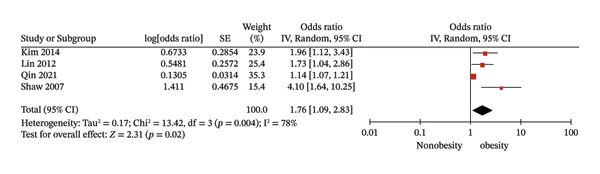
Forest plot of the association between obesity and albuminuria.

**FIGURE 3:**
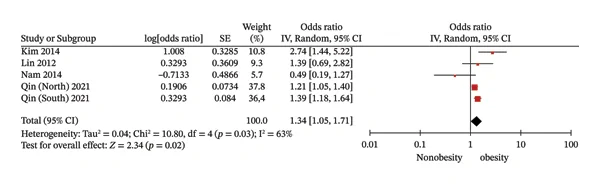
Forest plot of the association between obesity and albuminuria in men.

**FIGURE 4:**
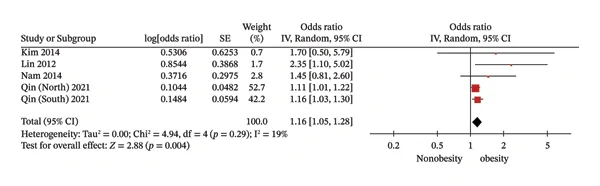
Forest plot of the association between obesity and albuminuria in women.

The pooled analysis showed an association between obesity and increased albuminuria (OR = 1.76; 95% CI: 1.09–2.83; *p* = 0.02). Nevertheless, a high degree of heterogeneity was detected among the studies (*I* ^2^ = 78%), implying that differences in study design, participant characteristics, or analytical methods may have influenced the variation in reported effects (Figure 2). Further analyses by sex reinforced this association. Obesity remained significantly linked to albuminuria in both men (OR = 1.34; 95% CI: 1.05–1.71; *p* = 0.02, *I* ^2^ = 63%) and women (OR = 1.16; 95% CI: 1.05–1.28; *p* = 0.004, *I* ^2^ = 19%), as shown in Figures 3 and 4. These results demonstrate a positive relationship between obesity and albuminuria across both sexes, despite the considerable heterogeneity between studies. All presented data were adjusted for potential covariates, as summarized in Table 2.

### 3.4. Sensitivity Analysis

Given the substantial heterogeneity observed in the pooled analysis (*I* ^2^ = 78%), sensitivity analyses were performed to assess the robustness of the findings and identify potential sources of heterogeneity. A leave‐one‐out sensitivity analysis was conducted by sequentially excluding each included study and recalculating the pooled effect estimate. The overall pooled OR remained stable regardless of which study was omitted, indicating that the association between obesity and albuminuria was robust.

Among the included studies, the study by Qin et al. contributed most substantially to the observed heterogeneity. Exclusion of this study reduced the heterogeneity from 78% to 25%, while the pooled association remained statistically significant. Compared with the other included studies, Qin et al. differed in participant characteristics, sample size, and the methods used to assess obesity. Specifically, obesity was evaluated using both BMI and waist‐to‐hip ratio (WHR) to represent general and central obesity, respectively. These methodological differences in obesity assessment may have contributed to the between‐study heterogeneity.

Overall, the sensitivity analysis demonstrated that the pooled effect estimates were consistent and statistically significant after the exclusion of each study. These findings indicate that the observed association between obesity and albuminuria is robust and not driven by any single study, despite the presence of substantial interstudy heterogeneity (Table 5).

**TABLE 5:** Leave‐one‐out sensitivity analysis.

Omitting	Pooled OR	95% CI	*I* ^2^(%)
Kim, et al.	1.73	0.96–3.14	80
Lin, et al.	1.85	0.95–3.61	82
Qin, et al.	2.11	1.40–3.19	25
Shaw, et al.	1.45	0.99–2.13	67

### 3.5. Confidence in Cumulative Evidence

According to the NOS‐CS, the included studies were generally of good methodological quality, indicating a low risk of major bias. All included studies consistently reported a positive association between obesity and albuminuria. Publication bias could not be formally assessed because fewer than 10 studies were included in the meta‐analysis. Imprecision was identified due to the relatively small sample sizes of several included studies. Furthermore, the certainty of the evidence was downgraded for serious inconsistency because of the substantial heterogeneity observed across studies evaluating albuminuria. No serious indirectness was identified, as the study populations, exposures, and outcomes were directly relevant to the review question.

Based on the GRADE framework, the overall certainty of the evidence was rated as low for most outcomes. However, the certainty of the evidence for the albuminuria outcome was rated as very low because it was downgraded for serious inconsistency and suspected publication bias, in addition to the inherent limitations of the observational study design (Table 6). Under the GRADE approach, evidence derived from observational studies is initially considered low‐certainty evidence; therefore, these additional limitations further reduced confidence in the estimated effect.

**TABLE 6:** GRADE evidence profile.

Outcome	Newcastle–Ottawa Scale (NOS)	Inconsistency	Quality assessment		Publication bias	Overall quality of evidence	Summary findings	
			Indirectness	Imprecision			Odds ratio total	95% CI (lower, upper)
Albuminuria	Not serious	Serious^a^	Not serious	Serious^b^	Not determined^c^	Very low	1.76	(1.09, 2.83)
Albuminuria (men)	Not serious	Not serious^a^	Not serious	Serious^b^	Not determined^c^	Low	1.34	(1.05, 1.71)
Albuminuria (women)	Not serious	Not serious^a^	Not serious	Serious^b^	Not determined^c^	Low	1.16	(1.05, 1.28)

^a^All studies reported that obesity increased the risk of albuminuria.

^b^Effect estimates come from a small number of studies.

^c^Publication bias was assessed qualitatively, and no unpublished studies were found in the literature search, thus not affecting the publication bias.

## 4. Discussion

This systematic review and meta‐analysis incorporated five observational studies with a total of 49,773 participants and found a positive association between obesity and increased odds of albuminuria in Asian populations. The pooled estimates indicated that people with obesity had nearly double the likelihood of albuminuria compared with those without obesity, supporting the role of obesity in the development of albuminuria. This relationship remained statistically significant in analyses stratified by sex, demonstrating a stable pattern in both men and women. Although considerable heterogeneity was present across studies, the overall evidence strengthens the link between excess adiposity and renal endothelial or glomerular damage and provides a basis for exploring potential mechanisms and clinical implications.

Previous investigations assessing abdominal obesity and albuminuria in diverse populations have reported mixed findings. Renal impairment in individuals with obesity is often attributed to coexisting T2DM or hypertension. Evidence from the Look AHEAD trial showed that higher BMI and central fat distribution were associated with albuminuria in overweight and obese adults with T2DM [18]. Comparable observations were reported in a large multicenter cross‐sectional study involving hypertensive participants from 26 countries [19].

The possibility that obesity itself contributes to kidney injury was first described in 1974 under the concept of obesity‐related glomerulopathy [20]. Subsequent large studies supported this notion, showing that central adiposity in nondiabetic individuals was linked to microalbuminuria and reduced renal function [21]. More recent research has likewise reported nearly a twofold increase in the risk of microalbuminuria among individuals with obesity [12]. Despite these findings, the exact biological pathways remain incompletely understood.

Several mechanisms have been proposed to explain how obesity may lead to albuminuria. Two commonly discussed pathways involve insulin resistance and hemodynamic alterations mediated through activation of the renin–angiotensin–aldosterone system (RAAS) [5]. A third pathway focuses on adipose tissue as an active endocrine organ. In obesity, increased fat mass leads to greater secretion of inflammatory mediators known as adipokines. Elevated levels of proinflammatory substances such as leptin, tumor necrosis factor‐α (TNF‐α), and interleukin‐6 (IL‐6), along with reduced circulating adiponectin, are frequently observed [22]. Lower adiponectin concentrations are strongly associated with insulin resistance and abnormalities in lipid and glucose metabolism [23]. Adiponectin also plays a key role in preserving the integrity of the glomerular filtration barrier. Animal studies have shown that adiponectin deficiency results in podocyte injury and increased albumin leakage, which can be partially reversed by adiponectin supplementation [24]. In vitro research further demonstrated that adiponectin reduces albumin permeability and oxidative stress in podocytes via activation of AMP‐activated protein kinase (AMPK). Additionally, adipose tissue contributes to RAAS activity, as adipocytes produce angiotensinogen and aldosterone, both of which are implicated in obesity‐related hypertension and renal stress [25].

Sex‐related differences may also influence the risk profile. The present findings indicate that obesity was positively associated with albuminuria in both men and women. However, earlier studies have reported inconsistent results. Data from the Framingham cohort suggested that visceral fat was associated with microalbuminuria in men but not in women [26]. In contrast, research in a Chinese adult population identified central obesity as an independent predictor among women [27]. These variations suggest that hormonal influences may play a role in modulating inflammatory responses. Differences in sex hormone levels, including the effects of 17β‐estradiol and its metabolites, have been proposed as potential contributors to these disparities [27].

The pooled analysis demonstrated substantial statistical heterogeneity (*I* ^2^ = 78%), indicating considerable variability among the included studies. Several methodological and clinical factors likely contributed to this heterogeneity. First, important differences existed in the assessment and definition of obesity. Some studies evaluated central obesity using WC or WHR, whereas others used BMI or imaging‐based measurements of visceral adiposity. These methods assess different aspects of adiposity and are not directly interchangeable. Imaging techniques provide more accurate estimates of visceral fat accumulation, while anthropometric measurements only indirectly reflect body fat distribution.

Second, the included populations differed considerably across studies. The studies involved participants from various Asian populations, including Korean, Chinese, Taiwanese, and South Asian cohorts. These populations possess distinct genetic predispositions, dietary patterns, metabolic profiles, and body composition characteristics. Asians are known to develop visceral adiposity and metabolic complications at lower BMI thresholds compared with Western populations, which may influence both the prevalence of albuminuria and the strength of the observed associations. There was considerable variation in the age of the included patients across the studies, which may have influenced the observed heterogeneity in the pooled results. However, subgroup analysis and meta‐regression were not feasible due to the small number of included studies.

Third, variability in inclusion and exclusion criteria may have contributed to heterogeneity. Some studies excluded participants with T2DM or hypertension, whereas others included general population samples with differing cardiometabolic risk profiles. Since albuminuria may arise through multiple mechanisms in diabetic and hypertensive individuals, differences in baseline comorbidities could influence effect estimates.

Fourth, methodological differences in study design and statistical adjustment may also explain the observed variability. Although most included studies used cross‐sectional designs, the covariates adjusted for in multivariable analyses differed substantially between studies. Variations in adjustment for age, sex, smoking, blood pressure, T2DM, and renal function may have affected the reported associations. This analysis demonstrated an association rather than temporal causation; therefore, whether obesity is a risk factor for albuminuria cannot be determined from this study.

Finally, differences in albuminuria assessment and outcome categorization may have further increased heterogeneity. Although most studies defined albuminuria using uACR thresholds, differences in laboratory techniques, cutoff values, and subgroup categorization reduced comparability across studies.

Despite substantial heterogeneity, sensitivity analyses showed that the association between obesity and albuminuria remained statistically significant after sequentially excluding individual studies, suggesting that the overall findings are relatively robust. Nevertheless, the pooled estimates should be interpreted cautiously, and future large‐scale prospective studies using standardized obesity and albuminuria measurements are needed to clarify the magnitude of this association.

## 5. Conclusion

This review suggests that obesity is positively associated with albuminuria in Asian populations. However, these findings are primarily representative of Asian cohorts and may not be generalizable to other populations with different genetic, environmental, and lifestyle characteristics. Therefore, further large‐scale, methodologically robust observational studies are needed to validate and strengthen these findings, as well as to determine their applicability across diverse populations in other regions.

## Funding

The authors received no funding for the study.

## Ethics Statement

This research does not involve human/animal.

## Consent

The authors have nothing to report.

## Conflicts of Interest

The authors declare no conflicts of interest.

## Data Availability

The data that support the findings of this study are available from the corresponding author upon reasonable request.
